# Mental health providers in Ukraine need support but they are not helpless: Professional self-organization and innovative practices

**DOI:** 10.3389/fpubh.2022.1009431

**Published:** 2022-11-04

**Authors:** Elena Cherepanov

**Affiliations:** Expert Consultant in Global Mental Health, Boston, MA, United States

**Keywords:** Ukraine, humanitarian emergency, war trauma, mental health needs, psychosocial response, professional self-organization, innovative practices

## Introduction

The Russia's military invasion to Ukraine has created a complex humanitarian emergency and the largest refugee crisis in Europe since World War II (WWII) (1). Complex emergencies tend to produce massive and urgent humanitarian and mental health (MH) needs and compromise access to the health care. Response places a heavy burden on local responders who are often members of the same community as the people they serve and are subjected to the same ailments (2). This paper calls for scaling up international support for MH professionals working in war zones and complex political contexts (3).

In this field report author reflects on the experience of remotely responding to a humanitarian crisis in Ukraine to draw preliminary conclusions on prioritized MH needs, available resources, and the barriers to accessing care. Understanding what has gone right may serve as an important lesson for MH providers and humanitarian agencies when planning responses to aiding Ukraine and the future emergencies.

Due to the ongoing armed conflict, verifiable data is scarce, it is hard to get, and many of my references are coming from personal sources, ubiquitous reports by my colleagues, and social media. Therefore, the results must be treated with caution. The situation in Ukraine remains politically complicated, and some responders chose against using their names.

## Mental health impact

The war trauma in Ukraine will likely have a long-term, possibly intergenerational MH impact (4, 5). Invasion came as a shock to many and felt like a *betrayal*. Few believed in a real possibility of an attack on the neighboring nation which shares deep historical, cultural, religious, and family ties. Reports about filtration camps, war rape, and kidnapping of Ukrainian children were interpreted as unambiguous evidence of genocidal intent by the Russia's government (6–9). The invasion has been unfolding in real time and has received great *media exposure*. The media attention has attracted the world's sympathy and mobilized worldwide support. Broad use of cell phones and social media has allowed people to stay connected and share vital information as it comes. Such informational overload has also, however, added to the confusion and created difficulty with finding reliable sources.

Based on my observations, providers' reports, and the analysis of the real time communications in the chat groups, the presenting problems have been changing with time. In the immediate aftermath, the most frequent complaints were about shock, disorientation, uncontrollable shaking, difficulties thinking clearly, and disbelief: “It's not real, it cannot be happening.”

Many reported symptoms of dissociation, intrusions, flashbacks, crying all the time, sleep problems and nightmares. Often symptoms persisted even after refugees reached a place of safety in neighboring countries. Because of the hypervigilance, people sometimes were refusing sleep medications and fought off falling asleep.

Gastrointestinal (GI) symptoms in refugees (stomach pain, nausea, and uncontrollable vomiting) were so prevalent and severe that, they invoked suspicion of poisoning. However, poison was ruled out by the International Red Cross workers, who suggested interpreting them as a stress reaction (10). GI stress reaction reminded me of the saying “sick to my stomach” which signifies a mixture of fear, repulsion and disgust. A similar expression exists in the Russian language: “*Toshno na dushe*,” “my soul is nauseated.” Previously, a strong link between GI symptoms, and trauma and anxiety has been found in war veterans (11), and also cross-culturally (12). For example, Cambodians may experience an “abdominal wind syndrome,” a GI syndrome associated with fear of attack (13).

By the end of March, when the reality started sinking in, depression, stresses of resettlement, and worries about the future came to the forefront. People were grieving personal losses, the loss of homes, and life before the war. Mental exhaust, emotional numbness, indifference, and disregard for their own lives, was seen in the refusals to go to shelters or to relocate to a safer place. Some struggled to control anger and rage. Increased irritability and family conflicts undermined family supports at a time when they are most needed. For many, the experience of fleeing and being separated from their family members was extremely traumatic especially when they had to make the decision about leaving behind enlisted men or frail family members.

Many children have witnessed shelling, destruction, and killings; some have been wounded, have lost, or been separated from their parents. Others witness to rape and torture. Parents reported to me and other providers the worsening of the conduct, traumatic attachment problems, and behavioral regression, i.e., acting younger than their age. Usually quiet and obedient children could become either clingy or defiant and act out an uncontrollable rage and anger, physically attacking their parents or peers. Other children presented with emotional detachment: they appeared “eerily quiet and apathic.” There were numerous reports of reoccurrence of enuresis in older boys and girls (personal communications with parents and schoolteachers in Ukraine and Poland).

More recently, psychosocial providers have noticed that somehow people “are getting used to the war,” and chronic or preexisting psychological problems such as family discord or problematic drinking have started to (re)surface (personal communication).

As it often happens during humanitarian crises, persons with special needs are especially vulnerable (14). They have fewer supports and encounter additional barriers to accessing already scarce resources.

Even with the extensive help of volunteers, the elderly, chronically ill, or persons with physical or mental special needs often were unable to relocate, access the health care, medications, and meet other basic needs. Some stayed behind because they were afraid to leave their familiar surroundings or did not want to be a burden to their family. They were not able to receive lifesaving medical treatment and have been suffering and dying of preventable causes.

## Strength and resilience

Russia's aggression has left citizens of Ukraine shocked and traumatized. It has also highlighted their resilience and strengthened national cohesion. Many named family, cultural values, faith, and their love for Ukraine as main sources of strength and inspiration for perseverance.

The *memories of surviving* WWII have been helping Ukrainians to survive the current war: elders, for example, have been teaching younger generations how to recognize the sounds of shelling, how to know when the danger is over, and where to find the safest places to hide.

In recent decades Ukraine has accumulated many well-trained MH professionals and crisis workers. A *widespread psychoeducation* increased the public's awareness of MH needs, educated about psychological trauma, and sensitized the public to recognizing trauma reactions. It helped raise awareness and destigmatize MH issues and formed a demand for trauma services.

In Ukraine, *the spirit of volunteering* has run exceptionally strong. Sometimes risking their own lives, volunteers have been checking on those in need, delivering food and medications. After relocation, refugees have immediately started volunteering themselves and sharing their resources. Many of those who earlier fled Belarus and Russia to escape political persecution have been helping with transportation and language services, babysitting, and connecting with health care.

## Professional self-organization

The initial MH response was marked by frantic and at times chaotic efforts by overworked MH responders to meet as many needs as possible. But only 2 months later, we saw an entirely different landscape in MH services provision. Although the systems of psychosocial support still face challenges, the professional community in Ukraine was able to come together in a very short time, overcome their differences, self-organize, and take charge in coordinating MH response, requesting specialized training and professional assistance when needed.

MH and psychosocial support providers in Ukraine, often traumatized and lacking safety themselves, have stepped in to provide psychological support and psychoeducation about coping with extreme stress and severe trauma. They have been bearing witness, advocating for the needs of the people they serve, and raising their voices against the war. The overall response to this humanitarian crisis has demonstrated the strong commitment of Ukrainian MH providers to their professional mission. This self-determination and the capacity to self-organize in response to pressing humanitarian needs indicates the maturation of a helping profession and helps to cement its role as an integral part of society.

## Barriers to accessing care

A tremendous amount of work has been done, and a lot has been accomplished, but many barriers remain. Finding bilingual resources has been a sensitive issue. Although the Russian language is commonly spoken in Ukraine, the war has understandably made a trigger for some Ukrainians.

Despite the widespread use of social media, many have not been aware of the available psychosocial resources or how to access them; vital information is spread mostly by word of mouth.

Ukrainian health and MH care systems had been functioning well-before the war. These systems were able to cushion most needs, but the availability of facilities, medications and logistics were compromised by the war.

Services offering has remained fragmented. Fragmentation in is an affliction that has traditionally hindered the effectiveness of humanitarian response. It adds to confusion, makes it difficult to navigate the system, and reduces efficiency in accessing scarce resources (15).

## Innovative practices: Telemedicine, helping chat groups, chatbots

### Telemedicine

Emerging technologies have transformed MH response to this emergency.

Accelerated digitalization of services during the COVID-19 pandemic has provided an unexpected advantage and expanded the access to MH practice and education. Telemedicine has allowed providing MH services remotely to communities that are otherwise difficult to reach, that are dispersed, or where it may not be safe to travel.

### Chat groups

Immediately after the invasion, the biggest challenge was connecting those in need with the providers. Almost instantaneously, existing virtual chats and support groups started accommodating requests from Ukraine and new ones started emerging on Telegram and Facebook. They aimed at real-time matching of on-site needs with local and remote resources. In these designated channels, anyone with war-related MH problems could post about their urgent needs, and self-appointed MH providers from different backgrounds – and often residing in different countries – would respond to these inquiries in real time.

The 2020 political crisis in Belarus led to the emergence of self-managed self-help chat groups. They have continued offering their capacity, MH resources, and the services to Ukrainians in need (e.g., https://www.facebook.com/groups/myrazam.info/). This model has proven itself useful in the aftermath of the invasion in Ukraine. Examples include: СвітлоЧат – Психологічна допомога українцям під час війни (Psychological help to Ukrainians during the time of war. Owner A. Pozhydayev), and Психологическая помощь Украина (Psychological assistance to Ukraine.).

As per owners' reports, at various times, these chat groups employed between 400 and 900 professional Ukrainian and Russian-speaking MH professionals from all around the world, but mostly from Ukraine. They responded to more than 40,000 inquiries. During busy days, they were receiving up to 1,000 inquiries, and currently receive about 200–300 requests a day.^1^ These chats were also used to share information about psychosocial resources and to provide peer support.

*Chatbots* in Ukrainian, Russian, and Belorussian languages are yet another innovative practice that is an example of growing transnational professional projects. For example, chatbots Faino (https://t.me/faino_psy_bot/relax$\@@underline\hbox{/}\mathsurround\z@$\relax) and GotoHelp (https://gotohelp.eu/). Although promising, further research into these innovative practices is needed before recommending their broader implementation.

## Conclusions and recommendations

Absent centralized guidance and often traumatized and unsafe themselves MH professionals in Ukraine have assumed responsibility for the psychological well-being of the community. In addition to providing care for the victims of atrocities, they have taken a lead in organizing community support and have become an inspiration and a driving force in shaping community resilience and recovery.

The response in Ukraine has demonstrated that public awareness of the psychological effects of trauma has strengthened community resilience and significantly reduced stigma of reaching out for MH help.

Lessons from this response underscored the importance of increasing trauma and Psychological First Aid competencies for MH and psychosocial providers, teachers, health care workers, and volunteers.

Many who most need aid will not reach out (14). The community outreach is needed to engage in services those who are least mobile, most vulnerable, severely traumatized, or depressed. Prioritizing the development of outreach workforce and the community-based support systems is essential.

The presence of the advanced health care systems and well-trained medical and MH cadres in Ukraine created unexpected challenge for humanitarian medical and psychosocial aid agencies. It highlighted the shortcomings of the traditional giving-receiving model based on the inherently in-built power differential (15). New models of psychosocial supports are needed. These models should be based on establishing equal partnerships and collaboration between foreign and Ukrainian professionals.

International professional community shall prioritize supporting local MH providers, which includes funding and technical support. In the spirit of humanitarian accountability, it is important that determination of the priorities, scope and scale of relief efforts is established in closed consultation with local providers who are to lead the response (15). The validity of the preconceived assumptions and approaches to crisis response and trauma treatment derived in a different context and culture must be carefully re-evaluated.

It is never too early to think about the future, even while war rages on. Although wars don't last forever, they do manage to sever communal, family, and professional ties. When the world is divided, MH professionals can play a key role in reminding both sides about humanitarian values, compassion, and caring—essential to building a peaceful future. Only then is there hope for a violence-free future.

## Author contributions

The author confirms being the sole contributor of this work and has approved it for publication.

## Conflict of interest

The author declares that the research was conducted in the absence of any commercial or financial relationships that could be construed as a potential conflict of interest.

## Publisher's note

All claims expressed in this article are solely those of the authors and do not necessarily represent those of their affiliated organizations, or those of the publisher, the editors and the reviewers. Any product that may be evaluated in this article, or claim that may be made by its manufacturer, is not guaranteed or endorsed by the publisher.

## Footnotes

^1^At the time when this paper was written.
